# PERfect Day: reversible and dose-dependent control of circadian time-keeping in the mouse suprachiasmatic nucleus by translational switching of PERIOD2 protein expression

**DOI:** 10.1111/ejn.16537

**Published:** 2024-09-19

**Authors:** David McManus, Andrew P. Patton, Nicola J. Smyllie, Jason W. Chin, Michael H. Hastings

**Affiliations:** Medical Research Council Laboratory of Molecular Biology, Cambridge Biomedical Campus, Cambridge, UK

**Keywords:** biological clock, Cryptochrome, period protein, synthetic biology, transcriptional inhibition

## Abstract

The biological clock of the suprachiasmatic nucleus (SCN) orchestrates circadian (approximately daily) rhythms of behaviour and physiology that underpin health. SCN cell-autonomous time-keeping revolves around a transcriptional/translational feedback loop (TTFL) within which PERIOD (PER1,2) and CRYPTOCHROME (CRY1,2) proteins heterodimerise and suppress trans-activation of their encoding genes (*Per1*,*2*; *Cry1*,*2*). To explore its contribution to SCN time-keeping, we used adeno-associated virus–mediated translational switching to express PER2 (tsPER2) in organotypic SCN slices carrying bioluminescent TTFL circadian reporters. Translational switching requires provision of the non-canonical amino acid, alkyne lysine (AlkK), for protein expression. Correspondingly, AlkK, but not vehicle, induced constitutive expression of tsPER2 in SCN neurons and reversibly and dose-dependently suppressed *pPer1*-driven transcription in PER-deficient (*Per1*,*2*-null) SCN, illustrating the potency of PER2 in negative regulation within the TTFL. Constitutive expression of tsPER2, however, failed to initiate circadian oscillations in arrhythmic PER-deficient SCN. In rhythmic, PER-competent SCN, AlkK dose-dependently reduced the amplitude of PER2-reported oscillations as inhibition by tsPER2 progressively damped the TTFL. tsPER2 also dose-dependently lengthened the period of the SCN TTFL and neuronal calcium rhythms. Following wash-out of AlkK to remove tsPER2, the SCN regained TTFL amplitude and period. Furthermore, SCN retained their pre-washout phase: the removal of tsPER2 did not phase-shift the TTFL. Given that constitutive tsCRY1 can regulate TTFL amplitude and period, but also reset TTFL phase and initiate rhythms in CRY-deficient SCN, these results reveal overlapping and distinct properties of PER2 and CRY1 within the SCN, and emphasise the utility of translational switching to explore the functions of circadian proteins.

## 1 Introduction

Daily rhythms of behaviour, sleep, and metabolism adapt mammals to the cycle of day and night (Nowak et al., 2021). These rhythms are driven by endogenous timing mechanisms, biological clocks, that are able to generate internal timing cues with periods of approximately 1 day, hence circadian. The principal circadian clock of mammals is the suprachiasmatic nucleus (SCN) of the hypothalamus (Hastings et al., 2018). Its autonomously generated circadian cycle of electrical activity is entrained to solar time by direct retinal innervation, and in turn, this rhythm of neuronal activity orchestrates the sleep/wake cycle (Collins et al., 2020) and associated physiological rhythms across all major organs (Brown & Azzi, 2013). Correspondingly, disruption of circadian order places a heavy toll on metabolic health (Brown, 2016).

Time-keeping in the SCN revolves around a cell-autonomous transcriptional/translational feedback loop (TTFL) within which PERIOD (PER1,2) and CRYPTO-CHROME (CRY1,2) proteins heterodimerise and suppress trans-activation of their encoding genes (*Per1*,*2*; *Cry1*,*2*) via E-box regulatory elements (Takahashi, 2017). The consequent alternation between accumulation of PER and CRY proteins during circadian daytime and their subsequent degradation during circadian night generates a self-sustaining negative feedback cycle with a period of ~24 h. Remarkably, the TTFL is active in almost all cell types across the body (Du & Brown, 2021), but when they are deprived of temporal cues from the SCN, synchrony is lost between different organs. Moreover, tissue-level oscillations progressively damp as individual cellular clocks within a tissue drift away from a common phase (Yoo et al., 2004). In contrast, when the SCN is isolated in organotypic slice culture, its cell-autonomous clocks exhibit high-amplitude, synchronised oscillations and the entire network maintains robust circadian cycles of electrical and metabolic activity, effectively indefinitely. These observations highlight the power of the autonomous SCN clockwork and the sophistication and extent of SCN-mediated circadian co-ordination across the organism. Indeed, when a functional TTFL is restored to the SCN alone, previously arrhythmic mutant mice exhibit robust and precise circadian rhythms of locomotor activity and sleep and show sleep-dependent memory comparable to wild-type mice (Maywood et al., 2021).

In the fly *Drosophila* and in the fungus *Neurospora*, the respective TTFLs have been modelled as a limit cycle (Leloup et al., 1999), a self-sustaining oscillation defined by “state variables”: molecular components that define circadian phase by their dynamic abundance (Lakin-Thomas, 1995). Whether the mammalian TTFL is a limit cycle and PER/CRY proteins act as state variables within it remain open questions (McManus et al., 2022), although PER2 is considered to be a nodal point of TTFL rhythmicity (Wilkins et al., 2007) (Chen et al., 2009) (D’Alessandro et al., 2015). When expressed constitutively, as opposed to rhythmically, the hallmarks of PER2 as a state-variable would be: an inability to initiate oscillation in an arrhythmic PER-deficient TTFL; suppression of endogenous PER2 and TTFL amplitude in wild-type SCN; lengthening of period, and the resetting of phase on an acute step-change reduction of PER2 levels. These criteria have been assessed and fulfilled in *Neurospora* and *Drosophila* through reversible, dose-dependent, and constitutive expression of candidate proteins from transgenes (Aronson et al., 1994) (Edery et al., 1994).

The aim of the current study was to explore the contribution of PER2 to SCN cell-autonomous time-keeping and its potential role as a state variable. To do so, the accompanying aim was to develop the method of translational switching in order to express PER2 constitutively and conditionally in organotypic SCN slices and then monitor the effects on the SCN clock. Translational switching exploits a cDNA encoding a protein of interest (in our case PER2) carrying an ectopic amber stop codon (TAG) (Chin, 2017). Under normal circumstances, this prevents translation of the protein when the cDNA is transfected into cells. The amber stop codon can be suppressed, however, by provision of an orthogonal tRNA that recognises the amber codon and is charged with a non-canonical amino acid (in our case alkyne lysine, [AlkK]) by a suitably engineered orthogonal tRNA synthetase (Ernst et al., 2016). Incorporation of the non-canonical amino acid into the peptide chain allows translational read-through and expression of full-length protein. Importantly, provision of different concentrations of AlkK and the ability to withdraw it make this manipulation tuneable and reversible. Previously, we have used translational switching to explore the role of CRY1 in the SCN TTFL (Smyllie et al., 2022) and to control circadian behaviour reversibly (Maywood et al., 2018). These studies indicate that CRY1 has some, but not all, properties of a state variable of the SCN TTFL insofar as its constitutive expression can increase the period and suppress the amplitude of the ongoing oscillation while its removal can set phase (McManus et al., 2022). To explore the role of PER2 in the SCN clock, and compare it with that of CRY1, we designed suitable constructs and adeno-associated viral (AAV) vectors to apply translational switching of PER2 in neurons in organotypic SCN slices.

## 2 Materials and Methods

### 2.1 Animals

All animal work was conducted under UK Home Office licence and overseen by the Animal Welfare and Ethical Review Body of the MRC Laboratory of Molecular Biology, under the Animals (Scientific Procedures) Act of 1986. Animals were bred and maintained at the LMB Ares biomedical facility on a 12:12 light–dark schedule. The original provenance of genetically altered mice is set out in Table S1.

### 2.2 Organotypic SCN slice preparation

SCN slices were prepared as described (Hastings et al., 2005). Both male and female mouse pups aged between P9 and P12 were culled and brain removed and quickly transferred to ice-cold GBSS (Sigma, USA) dissection medium (5 mg/mL glucose, 100 nM MK-801 (Thermo Fisher Scientific, USA); 3 mM MgCl_2_ and .05 mM AP5, (Sigma)). Coronal brain slices (300 μm) were prepared on a Mcllwain (U.K.) tissue chopper, and those containing SCN tissue were trimmed to remove any extraneous non-SCN tissue. Slices were transferred to membrane inserts (Millipore, USA) sitting on top of tissue culture medium (50% Basal Medium Eagle (BME) (Sigma); 25% Earle’s balanced salt solution [EBSS] (Sigma); 25% heat inactivated horse serum (Invitrogen, USA); 5 mg/mL D-glucose; 25 μg/mL Penicillin/Streptomycin; 1% Glutamax (Invitrogen); pH 7.2, osmolarity 315–320 mOsm), supplemented with 100 nM MK-801 (Thermo Fisher Scientific), 3 mM MgCl_2_ and .05 mM D-AP5 (Sigma), to block excitotoxicity. After 2–4 h, slices were transferred to 6-well dishes with fresh medium, without MK-801, MgCl_2_ or AP5, for long-term culture (37°C, 5% CO_2_).

### 2.3 Bioluminescence and multiplexed bioluminescence/fluorescence recordings

Slices were transferred to 35 mm dishes containing 1.2 mL recording medium (Dulbecco’s Modified Eagle Medium (D-MEM) (Sigma); .35 mg/mL NaHCO_3_ (Thermo Fisher Scientific); 5 mg/mL glucose; 25 μg/mL Penicillin/Strepto-mycin; .01 M HEPES (Invitrogen)) further supplemented with foetal calf serum (Gibco, USA), B27 (Gibco), Glutamax (Invitrogen) and 10 μM Luciferin (Biosynth, Switzerland), sealed with a cover-glass and silicone grease. For bioluminescence-only recordings, luciferase bioluminescence was detected by a photon multiplier tube (PMT; Hamamatsu, Japan), or Lumicycle (Actimetrics, USA), maintained at 37°C in a light-tight incubator. Photon counts were recorded every second, and counts were combined in 6-minute bins. To obtain a read-out of transcriptional rhythms of the core TTFL, bioluminescence was recorded from transgenes encoding luciferase driven by regulatory sequences from either the *Per1* promoter (*pPer1*) (Yamaguchi et al., 2003) or the *Cry1* promoter (*pCry1*) (Maywood et al., 2013). A read-out of translational rhythmicity of the core TTFL was obtained by recording bioluminescence from the PER2::Luciferase knock-in construct, where PER2 protein is fused at its C-terminus to luciferase (Yoo et al., 2004). For multiplexed bioluminescence/fluorescence recordings, slices were transferred to glass bottomed 35 mm dishes (Mattek, U.S.A.) containing 1.2 mL recording medium before being placed onto the heated stage of an LV200 microscope (Olympus, Japan) as described (Patton et al., 2023). Neuronal calcium levels were monitored by the human *Synapsin1* (*pSyn1*)-driven GCaMP6f or NES-jRCaMP1a reporters delivered via AAVs. Bioluminescence and fluorescence were acquired at 30-min intervals. Exposure times were between 9.0 and 29.5 min depending on specific LV200 setup for bioluminescence and 100 ms for fluorescence. Calcium fluorescence was detrended by fitting a linear trend to the recording to account for the rising baseline caused by constitutive *pSyn* activity increasing reporter levels, and bioluminescence and fluorescence traces were smoothed using a 2.5-h moving average (McManus et al., 2022). Circadian parameters were analysed as described below.

#### 2.3.1 Viral transduction of SCN slices

AAV design and production details are set out in Table S2. SCN slices were transduced with AAVs after a medium change (culture medium or recording medium, depending on the experiment) immediately prior to transduction. Slices received 1 μL of AAV with titre of at least 1 × 10^13^ GC/mL directly onto them and were incubated for 7 days before transfer into fresh medium.

#### 2.3.2 Translational switching

The translational switching system relies on the ability of Pyrrolysyl tRNA synthetase (PylRS) derived from *Methanosarcina* sp., and its cognate tRNA^Pyl^_CUA_ (PylT) to incorporate an amino acid at the amber stop codon (Chin, 2017). Through directed evolution, PylRS derivatives have been evolved to charge PylT with the non-canonical amino acid Alkyne Lysine (AlkK: N6-2-propynyloxycarbonyl-l-lysine). Expression of the orthogonal tRNA synthetase/tRNA pair allows read-through across the amber stop codon so long as AlkK is provided. PylRS and PylT can be expressed in organotypic SCN slices following delivery by AAVs. PylRS and PylT are packaged as part of the same expression plasmid in the same AAV to ensure transduced cells express both the synthetase and the tRNA. Selection of the promoter driving PylRS expression provides a second layer of control to decide which cells express the orthogonal tRNA Synthetase/tRNA pair. Using the *pSyn1* promoter ensures PylRS expression is restricted to SCN neurons and is constitutive (McManus et al., 2022). The construct also contains regulatory elements such as poly-adenylation sites (hGH pA) and woodchuck hepatitis virus posttranscriptional regulatory element (WPRE) to facilitate high levels of expression, along with a fluorescent protein to confirm successful expression that is typically co-expressed with PylRS. To ensure the fluorescent protein does not interfere with the function of PylRS, they are separated by a 2A peptide (P2A) that induces ribosomal skipping during translation resulting in the production of two separate proteins from the same mRNA. Delivery of PylRS/PylT and cDNA of a protein of interest containing an amber insertion within its coding sequence into organotypic SCN slices via transduction with separate AAVs allows translational switching of the target protein dependent on provision of AlkK to the media.

SCN slices were sequentially incubated with each of the two AAVs for 7 days separated by a medium change with fresh culture medium. Transduction with the AAV encoding PylRS/PylT was always performed first, followed by transduction with the AAV containing tsPER (TAG). Where the fluorescent calcium reporter was required, transduction with its AAV was performed before the AAV containing the orthogonal synthetase. To allow translation of tsPER (TAG), AlkK was added to the recording medium with a final concentration of 1–10 mM. Removal of AlkK was achieved by six successive 15 min wash-out exchanges with fresh recording medium.

### 2.4 SDS-polyacrylamide gel electrophoresis and Western blotting

HEK293T cells were grown in 6-well culture dishes and transiently transfected with plasmids and subsequently lysed in ice-cold RIPA Lysis and Extraction Buffer (Thermo Fisher Scientific) supplemented with Complete Protease Inhibitor (Roche, Switzerland). Lysates were separated at 200 V for 60 min using 4–12% Bi-Tris NuPage gels (Invitrogen) and then transferred onto a nitrocellulose membrane using the Trans-Blot Turbo Transfer System (BioRad, USA). Membranes were blocked in a 5% milk tris-buffered saline supplemented with Tween (TBS-T) solution (10 mM Tris (pH 8.0), 150 mM NaCl and .05% Tween-20) for 30 min at room temperature. Membranes were then incubated in anti-HA antibody (ab20084) TBS-T solution for 2 h at room temperature. After three 5 min washes in TBS-T, membranes were incubated for 1 h at room temperature in a 1:2000 goat anti-rabbit IgG HRP-linked antibody (ab6721) TBS-T solution. After three further washes, protein was detected using Amersham ECL Western blotting detection reagents (GE Healthcare, U.K.) and visualised using Chemidoc MP Imaging System (BioRad).

### 2.5 Immunostaining and imaging of cells and SCN slices

After washing in phosphate-buffered saline (PBS), HEK293T cells to be immunostained were incubated in buffer (1% BSA, .3% Triton-X in PBS) with 5% goat serum for 1 h to reduce non-specific background, and then incubated in a primary antiserum solution (Anti-HA tag anti-body (ab20084)) overnight at 4°C. The following day, after two washes, cells were incubated for 1 h at room temperature in secondary Alexa fluor-conjugated antisera. Cells were then washed twice in immunostain buffer, twice in PBS, and mounted on glass slides. Vectashield mounting medium without DAPI (Vector Laboratories, UK) was used if the sample contained blue fluorescence. Where native fluorescence was imaged, SCN slices were fixed in 4% paraformaldehyde for 30 min before three washes with PBS. The Millipore membrane was then cut to reduce size. SCN were incubated with primary, then secondary antisera solutions and mounted as described above. Cells and SCN slices were imaged using Zeiss LSM710, 780 or 880 systems using a 63× oil immersion apochromatic objective. Further processing was carried out within FIJI (Schindelin et al., 2012).

### 2.6 Circadian statistics

Rhythmicity and arrhythmicity were determined by calculating the autocorrelation of recordings that were detrended by fitting a second-order polynomial function at a lag corresponding to the cycle period. Autocorrelation was calculated in R using the acf() function in the base R stats package. To calculate any change in the level of bioluminescence in arrhythmic PER-null traces, the pre-treatment level of transcription was calculated as the mean of bioluminescence recordings taken 48 h immediately before treatment. To determine any acute suppression, we identified the local minimum of bioluminescence that appeared between 6 and 30 h after treatment with AlkK. For vehicle-treated slices where no such minimum existed, the bioluminescence was meaned across the interval of 24 h spanning 6 to 30 h following treatment. For each slice, the calculated level of bioluminescence was expressed as a ratio of the pre-treatment value. To measure any longer-term suppression of bioluminescence, the mean level of bioluminescence in an interval of 24 h starting at the point exactly 4 days following treatment was calculated and again expressed as a ratio of the mean of bioluminescence 48 hours immediately before treatment.

Circadian parameters in rhythmic SCN data were calculated by identifying peaks and troughs in the data as local minima and maxima (McManus et al., 2022). From these data, peak times could be used to derive the period on a per-cycle basis as the difference in timing between consecutive peaks. The average period was taken as the average of at least 3 of these peak-to-peak periods. Period estimates were confirmed by the fast Fourier transform–linear non-least Squares (FFT-NLLS) within the BioDare (www.biodare2.ed.ac.uk) circadian rhythms analysis software package (Zielinski et al., 2014). The first 24 h after any treatment were excluded from determination of period by BioDare. Amplitude was calculated on a percycle basis as the difference in bioluminescence signal between peak and the average of the troughs flanking the peak. Because brief perturbation of the organotypic SCN slices can result in temporarily elevated levels of bioluminescence that can mask the ongoing circadian oscillation, calculations took the first trough reached between 6 and 30 h after treatment as the start of the first relevant oscillation. Amplitudes were then normalised to the last cycle before treatment. Acute and sustained changes in amplitude were assessed on the first and fourth valid cycles post-treatment, respectively.

For group data, the circular mean, Rayleigh tests, and 95% confidence intervals were calculated and plotted using the mean circular, Rayleigh test and plot circular functions using the “circular” package in R (Agostinelli & Lund, 2023); mean vectors were added to the circular plots manually. To compare the circadian phase of independent slices, the peak of emission from PER2::Luciferase SCN was defined as CT12 (Brancaccio et al., 2013). The peak of *pCry1*-Luciferase emission is later than this (Fustin et al., 2009), although combined recording of PER2::Luciferase and *pCry1*-Luciferase recordings to phase-map them is not possible because their bioluminescent signals cannot be segregated. We therefore phase-mapped the *pCry1*-Luciferase peak against the rhythm of SCN neuronal intracellular calcium ([Ca^2+^]_i_) reported by GCaMP6f (n = 3), which peaks at CT7.6 (Patton et al., 2020) or jRCaMP1a (*n* = 3), which peaks at CT6.0 (Patton et al., 2023) in combined bioluminescence and fluorescence recordings. This identified the *pCry1*-Luciferase peak at CT13.5 ± .3 h (*n* = 6). This was used to account for the differential phasing of PER2::Luciferase and *pCry1*-Luciferase and register all slices in circadian time (McManus et al., 2022).

## 3 Results

### 3.1 Design and validation of constructs for translational switching of PER2

**Figure 1:**
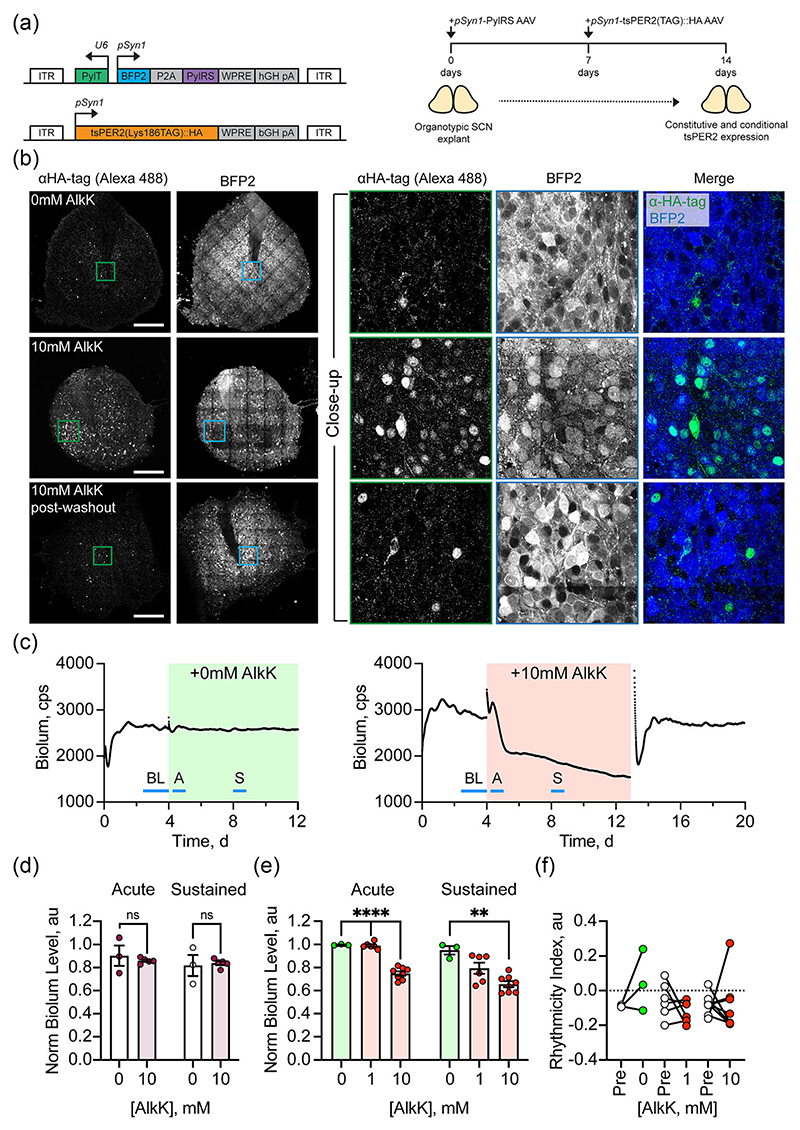
Translational switching of PER2 inhibits transcriptional activity in PER-null SCN carrying a *Per1 E-box-Luciferase* reporter. (a) (Left) Schematic view of constructs delivered by AAV vectors to achieve translational switching of PER2 (tsPER2). PylRS: Pyrrolysyl tRNA synthetase, PylT: tRNA^Pyl^_CUA_: BFP2: blue fluorescent protein 2: P2A: 2A cleavable peptide: WPRE: woodchuck hepatitis virus post-transcriptional regulatory element: bGH pA: growth hormone poly-adenylation site: ITR: viral inverted terminal repeat. (Right) Typical experimental time-line to generate slices capable of constitutive and conditional tsPER2 expression. (b) Confocal images of organotypic SCN slices confirm reversible, AlkK-dependent expression of tsPER2 following transduction with AAVs as in (a). Left two columns: low power views of anti-HA immunoreactivity (left) and BFP2 signal (right) from AAV encoding tRNA synthetase. Right three columns: high power views of boxed areas in low power images, presented as single channels and merged image. Upper row: Vehicle-treated SCN; middle row: SCN treated with 10 mM AlkK; lower row: SCN 12 h after removal of AlkK. Scale bar is 250 μm. (c) Representative traces of bioluminescence from PER-null SCN carrying a *pPer1* E-box-Luciferase reporter, transduced with AAVs as in (b) and treated chronically with either vehicle (left, green shading) or 10 mM AlkK (right, red shading). The lines mark the points used to calculate the pre-treatment baseline (BL), acute (A) and sustained (S) responses to treatment. (d) Group data showing acute (left) and sustained (right) levels of bioluminescence in untransduced PER-null SCN treated with vehicle or 10 mM AlkK. ns: no significant difference by Mann–Whitney *U*-test, *U* = 5, n1 = 3, n2 = 5, *P* = 0.57 and *U* = 6, n1 = 3, n2 = 5, *P* = 0.79. (e) Group data showing acute (left) and sustained (right) levels of bioluminescence in AAV-transduced PER-null SCN treated with vehicle, 1 mM or 10 mM AlkK. Treatment effect, Brown-Forsythe ANOVA: F*_(2,11.07)_ = 129.6, *P* < 0.0001 and F*_(2, 10.04)_ = 13.28, *P* = 0.002, with Dunnett’s T3 post-hoc test. (f) Rhythmicity index of bioluminescence of PER-null SCN, before (pre-) and during treatment with vehicle or AlkK. Two-way ANOVA: time *F*_(1, 14)_ = 0.23, *P* = 0.64, treatment *F*_(2, 14)_ = 1.27, *P* = 0.31, interaction *F*_(2, 14)_ = 1.46, *P* = 0.27. Points represent individual SCN with paired measures joined. Bars represent mean ± SEM. Significance: *P < 0.05, **P < 0.01. AAV, adeno-associated virus; AlkK, alkyne lysine (N6-2-propynyloxycarbonyl-l-lysine); ANOVA, analysis of variance; PER2, period 2; SCN, suprachiasmatic nucleus; SEM, standard error of the mean.

The ideal site for amber incorporation is at a lysine codon sufficiently N-terminal to ensure that any truncated peptide expressed in the absence of AlkK would not be functional. PER2 contains PER-ARNT-SIM (PAS) dimerisation domains required for homo- and hetero-dimerisation (Hennig et al., 2009), mutations of which disrupt circadian rhythms (Militi et al., 2016). Two suitable sites for amber incorporation were identified, Lys186 and Lys216, within or N-terminal to the PAS-A domain (Suppl. Figure S1). cDNA plasmid constructs carrying amber codons at one or other site, and with an EGFP C-terminal tag on PER2 were co-transfected into HEK293T cells with the requisite tRNA and tRNA-synthetase (Suppl. Figure S2A). The human Synapsin1 (*pSyn1*) promoter was chosen to ensure that once delivered via AAV into organotypic SCN slices, the expression of tRNA and tRNA-synthetase would be restricted to SCN neurons. In the absence of AlkK, only fluorescent red signal was evident from the mCherry encoded with the tRNA-synthetase, confirming activity of the *pSyn1* promoter. There was no detectable green EGFP signal from the C-terminal tag of tsPER2 (Suppl. Figure S2B, C). Provision of 10 mM AlkK triggered expression of EGFP from both constructs, indicative of translational read-through and expression of full length tsPER2::EGFP. To facilitate subsequent packaging of cDNA encoding tsPER2 within an AAV vector for use in the SCN, the EGFP fusion with tsPER2(Lys186TAG) was replaced by an HA tag in the plasmid and its expression in HEK293T tested by immunofluorescence and western blot. In transfected cells not treated with AlkK, mCherry signal was extensive but HA-immunoreactivity (HA-ir) was only detected in a few sporadic cells (Suppl. Figure S2D). Provision of 10 mM AlkK triggered abundant expression of HA-ir, indicative of the translation of full length tsPER2::HA. Furthermore, the effect was reversible as signal declined on removal of AlkK, post-washout. Western blot was used to confirm the expression of full length tsPER2::HA in cell extracts (Suppl. Figure S2E). A positive control for HA-ir was provided by transfection with a construct encoding HA-tagged casein kinase 2, which revealed strong expression. In the cells transfected with the synthetase and tsPER2 (Lys186TAG), but not treated with AlkK, no tsPER2::HA was evident, whereas in cells treated with AlkK, an appropriately sized immunoreactive band was evident at ~130 kDa, indicative of AlkK-dependent expression of full-length tsPER2::HA. The effective construct was then packaged into AAV and co-transduced along with an AAV encoding the tRNA and tRNA-synthetase (with a blue fluorescent protein [BFP] reporter) into SCN organotypic slices (Figure 1(a)). After 7 days, the signal from the *pSyn1*-driven BFP confirmed effective transduction, but in the absence of AlkK, there was only a sporadic immunoreactivity to HA (Figure 1(b)). In SCN treated with 10 mM AlkK, however, strong nuclear immunoreactive signal reported the extensive *pSyn1*-driven expression of full length tsPER2::HA. Moreover, the abundant nuclear signal had fallen dramatically 12 h after removal of AlkK, as levels of tsPER2::HA declined. These results confirm the efficacy of the constructs for reversible translational switching of constitutive PER2 expression.

### 3.2 tsPER2 as a negative regulator of SCN TTFL transcription

To test the contribution of PER2 to transcriptional negative feedback in the SCN TTFL, bioluminescent emission was recorded from *Per1*,*2*-null SCN carrying a transgenic *pPer1 E-box*-Luciferase reporter (Yamaguchi et al., 2003). The SCN were either left untransduced or were transduced with AAVs for translational switching. Before any further treatment, *pPer1*-driven bioluminescence was arrhythmic, as anticipated (Maywood et al., 2014) (Figure 1(c)). In untransduced SCN, addition of vehicle had no acute effect on bioluminescence when compared with the pre-treatment state (Figure 1(d)). Moreover, in untransduced SCN, 10 mM AlkK had no significant acute effect on bioluminescence levels compared to vehicle treatment. In transduced SCN, addition of vehicle again had no significant effect when compared with the pre-treatment state (Figure 1(c,e)). In contrast, treatment with AlkK caused an acute and dose-dependent suppression of *pPer1*-driven bioluminescence, with significant suppression by 10 mM but not 1 mM AlkK (Figure 1(e)). With sustained treatment, neither vehicle nor 10 mM AlkK had an effect on untransduced SCN (Figure 1(d)). In transduced SCN, however, AlkK caused a dose-dependent suppression of bioluminescence, with 10 mM being effective. On removal of AlkK by medium change (wash-out), levels of bioluminescence increased, indicative of clearance of tsPER2 (Figure 1(c)). The constitutive expression of functional tsPER2 did not, however, initiate rhythmicity in the PER-deficient SCN (Figure 1(c)), the rhythmicity index remaining very low and unchanged under 10 mM AlkK, despite the observed transcriptional inhibition (Figure 1(f)). The constructs therefore allowed dose-dependent and reversible constitutive expression of tsPER2, and revealed its role in transcriptional inhibition, but its inability to rescue rhythmicity in PER-null SCN when expressed constitutively.

### 3.3 tsPER2 regulates amplitude and period of the SCN TTFL

**Figure 2:**
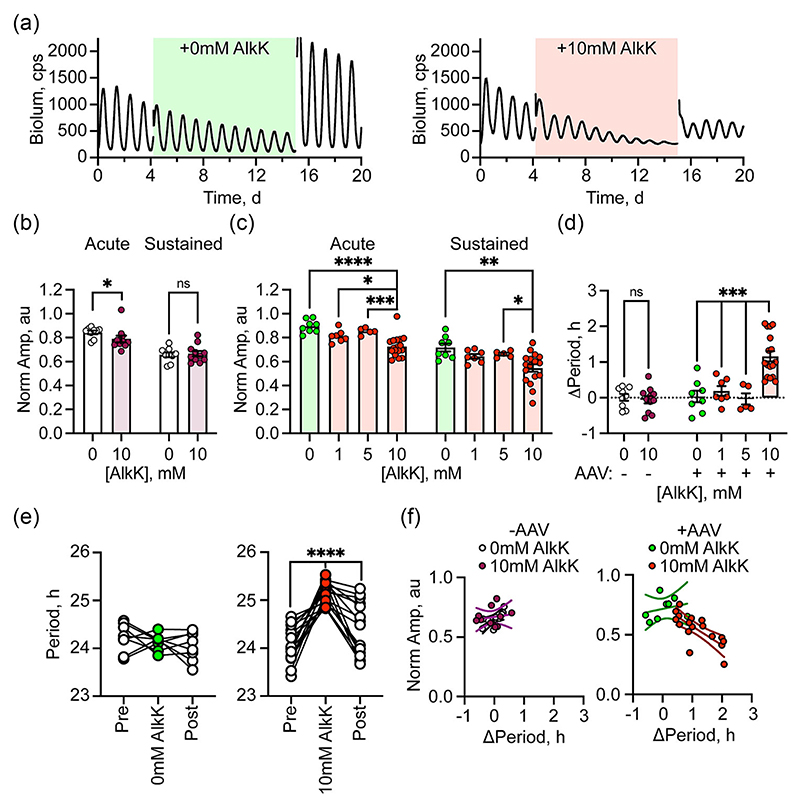
Translational switching of PER2 reversibly and dose-dependently damps the amplitude and prolongs the period of the circadian clock of the SCN, as reported by PER2::Luciferase. (a) Representative PER2::Luciferase bioluminescence traces of SCN slices transduced with AAVs for translational switching and treated with vehicle (left, green shading) or 10 mM AlkK (right, red shading) followed by washout with fresh medium. (b) Group data showing the acute and sustained effects of treatment with either vehicle or 10 mM AlkK on the amplitude of the PER2::Luciferase bioluminescence rhythm of untransduced SCN. * Mann–Whitney *U*-test, *U* = 18, n1 = 9, n2 = 11, *P* = 0.02, ns: no significant difference Mann–Whitney *U*-test *U* = 49, n1 = 9, n2 = 11, *P* = 0.99. (c) Group data showing acute and sustained effects of treatment with either vehicle or AlkK on the amplitude of the PER2::Luciferase bioluminescence rhythm of AAV-transduced SCN. One-way Brown-Forsythe ANOVA (acute) F*_(3,31.68)_ = 19.25, *P* < 0.0001, with Dunnett’s T3 post-hoc test: (sustained) F*_(3,27.58)_ = 9.79, *P* = 0.0001, with Dunnett’s T3 post-hoc test. (d) Group data showing effect of vehicle or AlkK on circadian period of untransduced (left) and AAV-transduced (right) SCN slices. Left: ns: no significant difference Mann–Whitney *U*-test *U* = 38.5, n1 = 9, n2 = 11, *P* = 0.42. Right: One-way Brown-Forsythe ANOVA left: *F*_(3,29.73_) = 19.52, *P* < 0.0001, with Dunnett’s T3 post-hoc test. (e) Circadian period of individual AAV-transduced SCN slices before, during, and after treatment with vehicle (left) or 10 mM AlkK (right) confirming reversibility of the effect of AlkK. One-way repeated measures ANOVA: vehicle: *F*_(2,14)_ = 1.09, *P* = 0.39: 10 mM AlkK: *F*_(2,32_) = 25.17, *P* < 0.0001, with Tukey’s post-hoc test. (f) Correlation between period lengthening and amplitude reduction (regression line and confidence limits) in untransduced (left) and transduced (right) SCN treated with vehicle (white, green) or 10 mM AlkK (purple, red). Only transduced SCN treated with AlkK exhibit a significant negative correlation (Pearson’s *r* = −0.73, *P* = 0.0009). In all plots, individual points represent individual SCN. Bars represent mean ± SEM. Significance: *P < 0.05, **P < 0.01, ***P < 0.001, ****P < 0.0001. AAV, adeno-associated virus; AlkK, alkyne lysine (N6-2-propynyloxycarbonyl-l-lysine); ANOVA, analysis of variance; PER2, period 2; SCN, suprachiasmatic nucleus; SEM, standard error of the mean.

To determine the effect of tsPER2 in SCN with an active TTFL, wild-type slices carrying the genomically encoded PER2::Luciferase translational reporter (Yoo et al., 2004) were left untransduced or were transduced with AAVs for translational switching. Before further treatment, all slices exhibited robust and precise rhythms of bioluminescence (Figure 2(a)). In untransduced slices, addition of vehicle had no significant acute effect on the amplitude of oscillation (Figure 2(b)). In contrast, the amplitude of the PER2:: Luciferase rhythm was significantly suppressed, compared to vehicle, by acute treatment with 10 mM AlkK. In SCN transduced for translational switching, acute treatment with vehicle had no significant effect on the ongoing oscillation, whereas treatment with 10 mM AlkK was followed by an acute fall of rhythm amplitude significantly different from vehicle (Figure 2(a,c)). The effect was dose-dependent insofar as 1 and 5 mM AlkK exhibited no significant change compared to vehicle (Figure 2(c)). This suggests, first, that AlkK may exert an acute effect independent to that of tsPER2, and second that AlkK-induced tsPER2 can additionally suppress the TTFL. The latter was clearly evident with the sustained effects of AlkK. Whereas 10 mM AlkK had no independent effect on rhythm amplitude in untransduced SCN (Figure 2(b)), in SCN transduced for translational switching it caused a significant damping of the amplitude of the PER2::Luciferase rhythm (Figure 2(c)). Again, this was dose-dependent, with 1 and 5 mM being without effect. This damping was due to reduced PER2::Luciferase expression at the oscillation peak because the trough of the oscillation did not change (Figure 2(a)). Furthermore, the quality of the rhythm was not affected by tsPER2 as indicated by the rhythmicity index (RI). For AAV-transduced SCN treated with vehicle, the RI was .55 ± .01 au and for SCN with oscillations damped by 10 mM AlkK, the RI of .56 ± .01 was not significantly different (repeated measures two-way analysis of variance: interval *F*_(1,22)_ = .02, *P* = .9; treatment *F*_(1,22)_ = 2.03, *P* = .2; interaction *F*_(1,22)_ = 2.85, *P* = .1). Therefore, despite the reduction in amplitude, the TTFL continued to oscillate with precision. Importantly, the effects of tsPER2 were reversible, with stable, non-damping oscillations restored by wash-out and removal of AlkK (Figure 2(a)).

Sustained, constitutively expressed tsPER2 therefore specifically, dose-dependently and reversibly damped the circadian oscillation of PER2::Luciferase bioluminescence. In untransduced SCN, treatment with either vehicle or 10 mM AlkK did not affect the period of the PER2:: Luciferase rhythm (Figure 2(d)). Similarly, treatment with vehicle of SCN transduced for translational switching had no effect. In contrast, treatment with 10 mM AlkK lengthened SCN period by >1 h (Figure 2(d)). Again, this was dependent on the dose of AlkK, being absent from AAV-transduced slices treated with 1 or 5 mM AlkK. Furthermore, the lengthening of period was fully reversible on wash-out (Figure 2(e)). Thus, the level of tsPER2 suppresses the peak expression of endogenous PER2 (i.e., PER2::Luciferase) and tunes the period of the SCN TTFL, both in a reversible manner. Indeed, the dual effects of tsPER2 induced by 10 mM AlkK were negatively correlated. In untransduced SCN treated with vehicle or AlkK and AAV-transduced SCN treated with vehicle, there was no significant relationship between sustained amplitude and period change (Figure 2(f)). In transduced SCN treated with 10 mM AlkK however, the slices that showed the greatest suppression of amplitude also showed the greatest period lengthening (Figure 2(f)). This relationship was not evident in untransduced SCN treated with 10 mM AlkK and further confirms a specific effect of AlkK-induced tsPER on the SCN TTFL.

### 3.4 tsPER2 regulates the period of the TTFL and SCN neuronal activity as reported by intracellular calcium [Ca ^2+^]_i_

**Figure 3:**
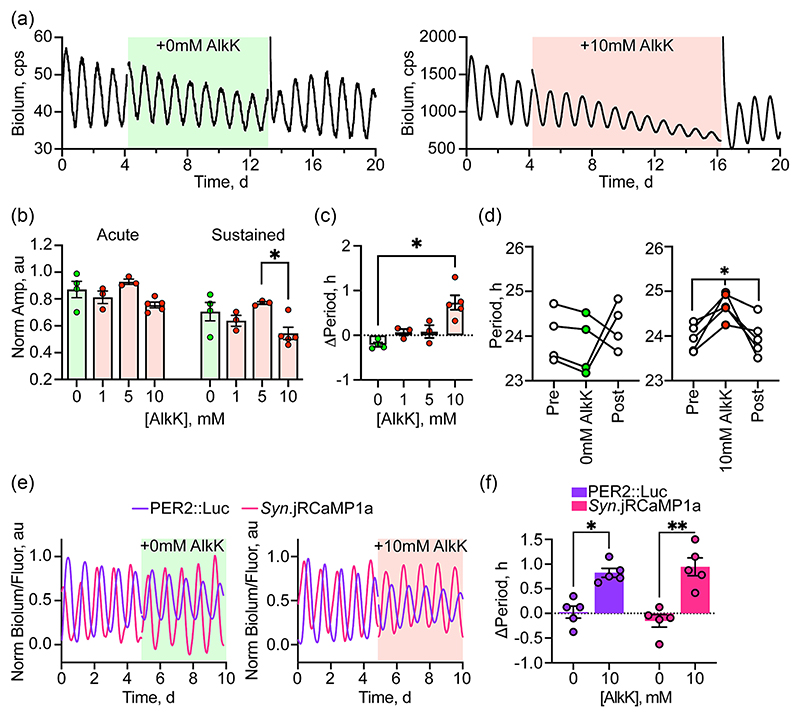
Translational switching of PER2 reversibly prolongs the period of the circadian rhythms of *pCry1-Luciferase* and intracellular calcium in the SCN. (a) Representative *Cry1*-Luciferase bioluminescence traces of SCN slices transduced with AAVs for translational switching and treated with vehicle (left, green shading) or 10 mM AlkK (right, red shading) followed by washout with fresh medium. (b) Group data showing (left) the acute and (right) the sustained effects of treatment with vehicle or AlkK on the amplitude of *Cry1*-Luciferase bioluminescence rhythm of AAV-transduced SCN. One-way Brown-Forsythe ANOVA: acute F*_(3,6.32)_ = 3.48, *P* = 0.09; sustained F*_(3,7.46)_ = 4.57, *P* = 0.04, with Dunnett’s T3 post-hoc test. (c) Group data showing dose-dependent effect of AlkK on circadian period of SCN slices. One-way Brown-Forsythe ANOVA F*_(3,7.58)_ = 14.02, *P* = 0.002, with Dunnett’s T3 post-hoc test. (d) Circadian period of individual SCN slices before, during and after treatment with vehicle (left) or 10 mM AlkK (right) confirming reversibility of the effect. One-way repeated measures ANOVA: vehicle: *F*_(2, 6)_ = 0.52, *P* = 0.61: 10 mM AlkK: *F*_(2,8)_ = 8.81, *P* = 0.01, with Tukey’s post-hoc test. (e) Representative traces of PER2::Luciferase bioluminescence and neuronal [Ca^2+^]_i_ from AAV-transduced SCN treated (shaded) with vehicle (left) or 10 mM AlkK (right). (f) Group data showing change in period of rhythms of PER2::Luciferase bioluminescence (left) and neuronal [Ca^2+^]_i_ (right) of SCN treated with vehicle of 10 mM AlkK. Repeated measures two-way ANOVA: treatment *F*_(1,4)_ = 69.71, *P* = 0.001, reporter *F*_(1,4_) = 0.10, *P* = 0.76, interaction *F*_(1,4)_ = 2.15, *P* = 0.22, with Sidak’s post-hoc test. In all plots, individual points represent individual SCN, with paired measures connected by lines. Bars represent mean ± SEM. Significance: *P < 0.05, **P < 0.01, ***P < 0.001. AAV, adeno-associated virus; AlkK, alkyne lysine (N6-2-propynyloxycarbonyl-l-lysine); ANOVA, analysis of variance; PER2, period 2; SCN, suprachiasmatic nucleus; SEM, standard error of the mean.

To test whether these effects of tsPER2 on the TTFL are generic rather than specific to the PER2::Luciferase reporter, the experiments were repeated on wild-type SCN carrying a transgenic transcriptional reporter in which a minimal *E-box* promoter from the *Cry1* gene drives luciferase expression (*pCry1*-Luciferase) (Maywood et al., 2013) (Figure 3(a)). With this reporter, and in contrast to the *pPer1*-Luciferase and PER2::Luciferase recordings, there was no significant acute effect of 10 mM AlkK on bioluminescence (Figure 3(b)). With sustained treatment, there was, however, an overall significant inhibitory effect, with bioluminescence amplitude being significantly reduced under 10 mM AlkK compared to 5 mM. Furthermore, the effects of tsPER2 on the period of the *pCry1*-Luciferase oscillation were directly comparable to those observed in PER2::Luciferase slices. Whereas treatment with vehicle had no significant effect on the ongoing circadian oscillation, treatment with 10 mM AlkK lengthened the period of the TTFL by nearly an hour, and the overall effect of AlkK was dose-dependent (Figure 3(c)). Furthermore, the effect was reversible on wash-out of AlkK (Figure 3(d)). Thus, regardless of the reporter used, the constitutive expression of tsPER2 lengthened the overall period of the SCN TTFL: by 1.2 ± .2 h in PER2::Luciferase slices and .73 ± .2 h in *Cry1*-Luciferase slices treated with 10 mM AlkK.

To test whether the effects of tsPER2 on the TTFL period carried forward on to the circadian rhythm of general neuronal activity, PER2::Luciferase SCN were transduced with AAVs for translational switching and also with an AAV encoding the fluorescent indicator of intracellular calcium ([Ca^2+^]_i_), jRCaMP1a, driven by the *pSyn* promoter. Treatment with vehicle had no effect on the ongoing oscillations of PER2::Luciferase bioluminescence or [Ca^2+^]_i_ (Figure 3e). In contrast, treatment with 10 mM AlkK caused a decline in the PER2::Luciferase bioluminescence amplitude, as noted above (vehicle, 38.4 ± 11.9% vs 10 mM AlkK, 62.8 ± 9.8% suppression at cycle 4, paired *t*-test *t*_[4]_ = 4.72, *P* = .009). More importantly, the addition of AlkK significantly lengthened the period of PER2 bioluminescence by .82 ± .09 hours, again as noted above (Figure 3(f)) and also significantly lengthened the period of the [Ca^2+^]_i_ rhythm to the same extent (.94 ± .18 h) (Figure 3(f)) and this was reversed on withdrawal of AlkK. Therefore, the period-setting effect of tsPER2 on the TTFL period was carried forward into the rhythm of general neuronal activity.

### 3.5 Acute reduction of tsPER2 levels does not reset phase of the SCN TTFL

**Figure 4:**
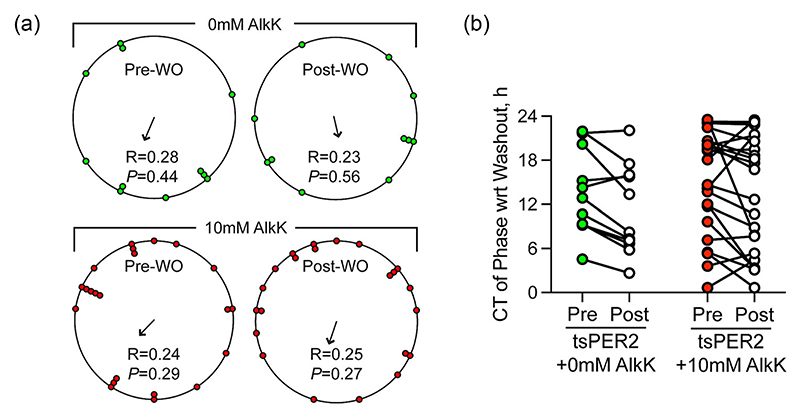
Acute withdrawal of tsPER2 does not alter SCN circadian phase. (a) Circular plots of circadian phase of SCN slices before (left) and after (right) wash-out (WO) of vehicle (top, green points) or 10 mM AlkK (bottom, red points). The mean phase is indicated by the direction of the arrow in the Centre, and the degree of synchrony is indicated by the vector length of the arrow (determined from R, the Rayleigh statistic which is also reproduced numerically on the plot). The *P*-value given in the plot is the significance of the *R* value. (B) Paired plots of SCN phase before and after wash-out of vehicle or 10 mM AlkK. AlkK, alkyne lysine (N6-2-propynyloxycarbonyl-l-lysine); PER2, period 2; SCN, suprachiasmatic nucleus.

As a final test of the role of PER2 in the SCN TTFL, the effect of acute withdrawal of tsPER2 on circadian phase was examined. The rationale was based on work with tsCRY1, which showed that when AlkK was washed out from a cohort of SCN slices to relieve the tsCRY1-dependent damping and period-lengthening of the TTFL, the formerly non-synchronous, independent SCN started to oscillate from a common phase defined by the point of wash-out. Consequently, their renewed oscillations were synchronous (McManus et al., 2022), showing that the acute step-change from high to low levels of tsCRY1 set the TTFL to a unique common phase. This protocol was repeated for tsPER2. Both PER2::Luciferase and *pCry1*-Luciferase SCN were transduced with AAVs for translational switching and circadian bioluminescence rhythms recorded before treatment with either vehicle (PER2::Luciferase: *n* = 8, *pCry1*-Luciferase *n* = 4) or 10 mM AlkK (PER2::Luciferase: *n* = 17, *pCry1*-Luciferase: *n* = 5). After 10 days of treatment and subsequent damping and period-lengthening of the oscillation, AlkK was removed from all slices by six changes of medium, allowing spontaneous oscillations to resume. Prior to washout, there was no synchrony between the independent SCN treated with either vehicle or AlkK and so wash-out occurred at a range of circadian phases across the independent SCN slices (Figure 4(a)). After medium change, the slices previously treated with vehicle oscillated in phase with pre-washout conditions, and consequently, there was no synchrony between slices (Figure 4 (a,b)). The SCN treated with AlkK and subject to suppression and lengthening of their TTFL by tsPER2 behaved in a manner similar to the vehicle-treated controls. The phase of oscillation after wash-out was not significantly different from the phase before wash-out, and there was no imposed synchrony between the slices (Figure 4(b)). The step-change in PER2 levels did not, therefore, set the TTFL to a new phase.

**Figure 5:**
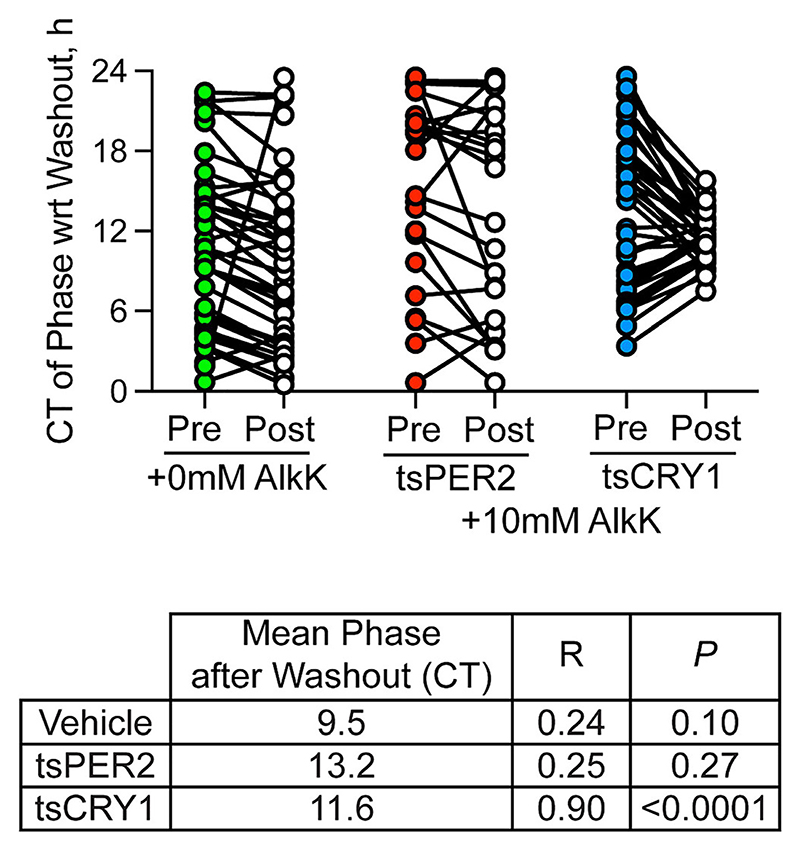
Meta-analysis of resetting actions of withdrawal of tsPER2 or tsCRY1 in SCN slices. Paired plots of SCN phase before (pre, left, coloured) and after (post, right, white) wash-out of vehicle (green) or 10 mM AlkK from SCN slices treated for expression of either tsPER2 (red) or tsCRY1 (blue). Rayleigh test statistics are given in the table below the plot indicating the mean phase in circadian hours, the Rayleigh statistic (*R*), and the *P*-value of the Rayleigh statistic for AAV-transduced slices previously treated with vehicle or with 10 mM AlkK to express tsPER2 or tsCRY1. Note that independent SCN slices were significantly synchronised by withdrawal of tsCRY1, whereas withdrawal of tsPER2 did not synchronise slices. AlkK, alkyne lysine (N6-2-propynyloxycarbonyl-l-lysine);PER2, period 2; SCN, suprachiasmatic nucleus.

As a positive control for phase-alignment, asynchronous PER2::Luciferase SCN were treated with the translational inhibitor, cycloheximide (CHX), to suspend the TTFL (Yamaguchi et al., 2003). On wash-out of CHX, the bioluminescence rhythm was restored and it was synchronous across the different slices, demonstrating that the damped TTFL had resumed its oscillation from the same phase in each slice (Suppl. Figure S3). This demonstrates that the failure to observe phase-alignment on withdrawal of tsPER2 was not caused by any technical aspect of the procedure. To provide further context for these results with tsPER2, they were compared with data from the comparable study of the effect of tsCRY1 on SCN TTFL phase as reported by PER2::Luciferase and *pCry1*-Luciferase bioluminescence (McManus et al., 2022). A meta-analysis of the composite data demonstrated the absence of synchrony for all slices across treatments before washout (Figure 5), and the continuing asynchrony after vehicle wash-out (post-washout *R* = .24, *P* = .10) or AlkK removal for tsPER2 (post-washout *R* = .25, *P* = .27). In contrast, withdrawal of tsCRY1 caused a pronounced phase-alignment of independent SCN (*R* statistic = .90, *P* < .0001) (Figure 5) as slices resumed oscillation from the phase defined by the step-change in levels of CRY1 (endogenous and tsCRY1).

## 4 Discussion

We designed constructs and AAV vectors to express translationally switchable PER2 in SCN neurons, driven by the constitutively active *pSyn1* promoter. In the absence of AlkK, expression of tsPER2::HA was sporadic and of low intensity. In contrast, provision and withdrawal of AlkK to and from the culture medium conferred dose-dependent and reversible control of extensive nuclear tsPER2 expression. This enabled us to test the control exerted by tsPER2 over a specific set of parameters of SCN time-keeping. In arrhythmic PER-deficient (*Per1*,*2*-null) SCN, constitutive expression of tsPER2 reversibly and dose-dependently suppressed *pPer1 E-box*-driven transcription, illustrating the potency of PER2 as a negative regulator within the SCN TTFL. Constitutive expression of tsPER2 failed, however, to initiate circadian oscillations in these arrhythmic SCN. In rhythmic, PER-competent SCN, AlkK dose-dependently reduced the amplitude of PER2::Luciferase oscillations as inhibition by tsPER2 progressively damped the TTFL. Alongside this, tsPER2 also dose-dependently lengthened the period of the SCN TTFL and neuronal calcium rhythms. Finally, following wash-out of AlkK to remove tsPER2, the SCN regained TTFL amplitude and period. Importantly, however, the SCN retained their pre-washout phase, i.e., the acute removal of tsPER2 from the damped oscillator did not phase-shift the TTFL. The current results emphasise the utility of translational switching to explore the functions of circadian (and potentially other) proteins by extending it to control the expression of a second negative regulator of the TTFL.

Previous analyses of PER2 function have successfully used transcriptionally based approaches, such as Tet-on/off, doxycycline switches (Chen et al., 2009) (I. T. Lee et al., 2015) (D’Alessandro et al., 2015). Here, we developed AAV-mediated translational switching as an alternative tool and applied it to neurons, although it can also be applied to astrocytes (Patton et al., 2023) and other cell types, by the selection of suitable promoters. One drawback of our approach is the limited packaging capacity of AAVs. This precluded the addition of a fluorescent tag to tsPER2, which could otherwise be used to monitor the dynamics of tsPER expression in real time. A further drawback may relate to the low-level HA-ir in SCN cells transduced with the AAVs. This may reflect either a low level of leaky expression at the amber stop codon and/or non-specific immunoreactive background signal. Again, direct visualisation in living cells of tsPER2 via a fluorescent tag could have informed this. It should be noted, however, that transduction of AAVs in the absence of AlkK did not lengthen SCN period compared to untransduced SCN and so any potential leakage was not material to TTFL function. Notwithstanding these considerations, it is evident that AAVs provide overall flexibility of approach for reversible expression of proteins by, for example, facilitating readily implemented changes to construct design, and do not require extensive intersectional breeding of mouse lines carrying genomic constructs.

A further methodological consideration is the potential for 10 mM AlkK alone to affect the SCN, although in untransduced *pPer1*-Luciferase SCN slices, treatment with AlkK alone had no acute or sustained effect on transcription. In rhythmic, untransduced PER2::Luciferase SCN addition of 10 mM AlkK alone did cause an acute damping of the oscillation but this was not sustained. In addition, in the effective condition of treating AAV-transduced SCN with 10 mM AlkK, there was a clear correlation between the extent of the two effects of tsPER2: damping and period-lengthening indicating specificity of action. Finally, treatment with 10 mM AlkK alone did not affect SCN circadian period. Therefore, whilst being cognisant of the potential confound of independent effects of non-canonical amino acids on the SCN, it is clear that AlkK-mediated induction of PER2 translation was causal in altering TTFL properties (Ernst et al., 2016).

Consistent with being a state variable, rhythmic expression of PER2 can rescue circadian behaviour in PER-null mice (D’Alessandro et al., 2015) whilst constitutive expression of PER2 failed to rescue PER-null SCN (present study). Furthermore, in mice and SCN with functional TTFLs, constitutive expression of PER2 damped and repressed the SCN TTFL (present study), and caused behavioural arrhythmicity in vivo (Chen et al., 2009) (I. T. Lee et al., 2015). Additionally, under circumstances where observable rhythmicity was sustained, circadian period was lengthened by constitutive expression, likely because the additional levels of PER2 require longer to be cleared by proteasomal degradation mediated by the balance of kinase and phosphatase activities (H. M. Lee et al., 2011) (D’Alessandro et al., 2015). This period lengthening is consistent with the effects of stabilising mutations in PER2 that correspondingly lengthen period (Masuda et al., 2020) along with destabilising mutations that shorten period (Militi et al., 2016). Indeed, mathematical modelling suggests that the period of the TTFL is set primarily by the PER2-based negative feedback loop (Wilkins et al., 2007). On the other hand, tsCRY1 can also lengthen SCN period (McManus et al., 2022) and genomic loss of either CRY1 or CRY2 shortens or lengthens SCN period, respectively (Anand et al., 2013), highlighting the role for CRY-mediated feedback in period-setting. These observations may be resolved because CRY1 regulates the stability and cellular localisation of PER proteins (Partch et al., 2014) (Smyllie et al., 2022). It may affect TTFL period, therefore, both indirectly by acting through the PER:CRY complex, and directly by acting independently of PER (Koike et al., 2012).

Comparisons of the effects of tsPER2 and tsCRY1 (McManus et al., 2022) emphasise this point (Table 1). This shows that they share a number of properties with regards to being a state variable, with both inhibiting their encoding genes, and their constitutive expression causes progressive damping of the TTFL and period lengthening. Nevertheless, there are also differences between their actions. For example, even though tsPER2 suppressed *pPer1 E-box*-Luciferase levels in PER-null SCN, it did not affect the nadir of PER2::Luciferase rhythms in wild-type SCN. In contrast, tsCRY1 not only suppressed PER2::Luciferase bioluminescence in CRY-null SCN but it also inhibited it in wild-type SCN, suppressing the baseline oscillation (McManus et al., 2022). Furthermore, expression of tsCRY1 lengthened period by ~2 h, whereas tsPER2 lengthened it by ~1 h. With the caveat that the same dose of AlkK may not have supported translation of CRY1 and PER2 in equimolar amounts, these results suggest, prima facie, that PER2 is less potent than CRY1 as a negative regulator in the SCN clock. A more marked difference, however, is seen with the control of SCN phase. The withdrawal of AlkK to stop production of tsPER2 and cause a step-wise reduction of total PER2 levels (endogenous PER2 plus tsPER2) did not shift the phase of the ongoing oscillation. In contrast, acute reduction of CRY1 levels through withdrawal of AlkK allowed the repressed TTFL to commence its full oscillation from a unique, defined phase. As noted above, it is possible that this contrast is a consequence of different levels of expression of tsPER2 and tsCRY1, but the tsPER2 was clearly active as a negative regulator and yet no sign of phase setting was obvious. This is surprising given that light-induced increased expression of *Per1* and *Per2* is the basis for entrainment of the SCN (Shigeyoshi et al., 1997), whereas *Cry* genes are not acutely responsive to light pulses (Field et al., 2000). Furthermore, non-photic cues that reset the SCN when applied during circadian day suppress *Per* expression in the SCN, leading to a phase advance in circadian behaviour (Maywood et al., 2002). To resolve these discrepancies, it will be necessary to image, in real-time, the dynamic changes in PER and CRY protein abundance both during the steady state oscillation and also when the TTFL has been perturbed, for example by addition and withdrawal of AlkK. Moreover, an alternative is to consider the PER:CRY complex as one entity, such that together these two proteins do fulfil the key criteria for them to be considered a state variable of the mammalian TTFL. Indeed, both PER2 and CRY1 proteins have been shown to occupy different intracellular pools, based on molecular mobility (Smyllie et al., 2022); thus, it would not be surprising if the behaviours of the proteins individually would be different from when they are in complex.

In conclusion, we have established translational switching as a means to express PER2 in SCN neurons in a reversible and dose-dependent manner. Using this method, we have shown both overlapping and distinct properties of PER2 and CRY1 within the circadian time-keeping mechanism of the SCN. Together, these results suggest that although individually lacking the full complement of properties, in combination, PER2 and CRY1 exhibit all of the necessary properties of a state variable of the proposed limit cycle oscillator of the SCN. This suggests that the PER2:CRY1 complex, rather than the individual proteins, operates as a state variable. Thus, the mammalian TTFL incorporates additional levels of complexity beyond those seen in *Neurospora* and *Drosophila*, where single state variables are active (Leloup et al., 1999).

## Supplementary Material

Table 1-2. Fig. 1-3.

## Figures and Tables

**Table 1:** Comparison of the properties of tsPER2 and tsCRY1 when expressed constitutively in SCN slices, colour-coded as consistent with (Y) or not consistent with (N) those of a state-variable of a putative SCN limit-cycle. Data for tsCRY1 from (McManus et al., 2022).

Property of state-variable	tsPER2	tsCRYl
Suppress SCN TTFL amplitude	**Y**	**Y**
Increase SCN TTFL period	**Y**	**Y**
Increase period of SCN neuronal activity rhythm	**Y**	**Y**
Unable to initiate TTFL in null-background	**Y**	**N**
Re-phase TTFL on withdrawal	**N**	**Y**

## Data Availability

All requests should be sent to the corresponding author. The datasets generated during and/or analysed during the current study are available from the corresponding author upon reasonable request.

## Abbreviations

AAV: adeno-associated virus
AP5: amino-5-phosphonovaleric acid
AlkK: alkyne lysine (N6-2-propynyloxycarbonyl-l-lysine)
BFP: blue fluorescent protein
bGH pA: bovine growth hormone poly-adenylation site
Bis-tris: 2,2-bis (hydroxymethyl)-2,2′,2″-nitrilotriethanol
BME: Basal Medium Eagle
BSA: bovine serum albumen
cDNA: complementary DNA
CHX: cycloheximide
CRY: Cryptochrome
CT: circadian time
DAPI: 4′,6-diamidino-2-phenylindole
D-MEM: Dulbecco’s Modified Eagle Medium
EBSS: Eagle’s balanced salt solution
EGFP: enhanced green fluorescent protein
FFT-NLLS: fast Fourier transform–linear non-least squares
GBSS: Gey’s buffered salt solution
HA-tag: human influenza hemagglutinin tag
HEPES: 4-(2-hydroxyethyl)-1-piperazineethanesulfonic acid
hGH pA: human growth hormone poly-adenylation site
HRP: horseradish peroxidase
IgG: immunoglobulin G
ITR: inverted terminal repeat
MK-801: 5-methyl-10,11-dihydro-5H-dibenzo[a,d]cyclohepten-5,10-imine
P2A: porcine teschovirus-1 2A peptide cleavage site
PAS: PER-ARNT-SIM
PER: period
PBS: phosphate-buffered saline
PMT: photon multiplier tube
PylRS: pyrrolysyl tRNA synthetase
PylT: tRNA^Pyl^_CUA_
RI: rhythmicity index
RIPA: radio-immunoprecipitation assay
SCN: suprachiasmatic nucleus
Syn: synapsin
TBS-T: tris buffered saline supplemented with tween
tRNA: transfer RNA
Ts: translational switching, translationally switched
TTFL: transcriptional/translational feedback loop
WPRE: Woodchuck hepatitis virus post-transcriptional regulatory element
[Ca^2+^]_i_: intracellular calcium concentration
-ir: immunoreactivity.

## Abbreviations

SCN: suprachiasmatic nucleus
TTFL: transcriptional/translational feedback loop.
