# Supplementary material for: PERfect Day: reversible and dose-dependent control of circadian time-keeping in the mouse suprachiasmatic nucleus by translational switching of PERIOD2 protein expression
Source: Eur J Neurosci. Author manuscript; Available in PMC 2024 Dec 9. (PMC7617102; doi:10.1111/ejn.16537)
Supplement: Table 1-2. Fig. 1-3. [file EMS201617-supplement-Table_1_2__Fig__1_3_.pdf]

Supplementary material, McManus et al.

Table S1. Animal Provenance

Strain	Alleles	Provenance
PER2::LUC	*mPer2*$^{Luc/Luc}$	(Yoo et al., 2004)
Cry1-Luc	*pCry1.Luc*	(Maywood et al., 2013)
Per1/2 null	*mPer1*$^{-/-}$, *mPer2*$^{-/-}$	(Bae et al., 2001)
Per1-Luc	*pPer1.Luc*	(Yamaguchi et al., 2003)

Table S2. AAV design and production details

**AAV full name**	**Construct Provenance**	**AAV Packaging**
AAV-pSyn1-mCherry	(Ernst et al., 2016)	Penn Vector Core
AAV-pSyn1-BFP2	(Ernst et al., 2016)	Penn Vector Core
AAV-pSyn1-PER2(186)TAG::HA	Plasmid designed and validated by DM. Plasmid synthesised and packaged by Vector Builder	Vector Builder
AAV-pSyn1-NES-jRCaMP1a-WPRE-SV40	pAAV-Syn-NES-jRCaMP1a-WPRE-SV40 was a gift from Douglas Kim & GENIE Project (Addgene plasmid # 100848 ; http://n2t.net/addgene:100848 ; RRID:Addgene_100848) (Dana et al., 2016)	Addgene
AAV-pSyn1-GCaMP6f-WPRE-SV40	pAAV.Syn.GCaMP6f.WPRE.SV40 was a gift from Douglas Kim & GENIE Project (Addgene plasmid # 100837 ; http://n2t.net/addgene:100837 ; RRID:Addgene_100837) (Chen et al., 2013)	Addgene

Supplementary Figure 1

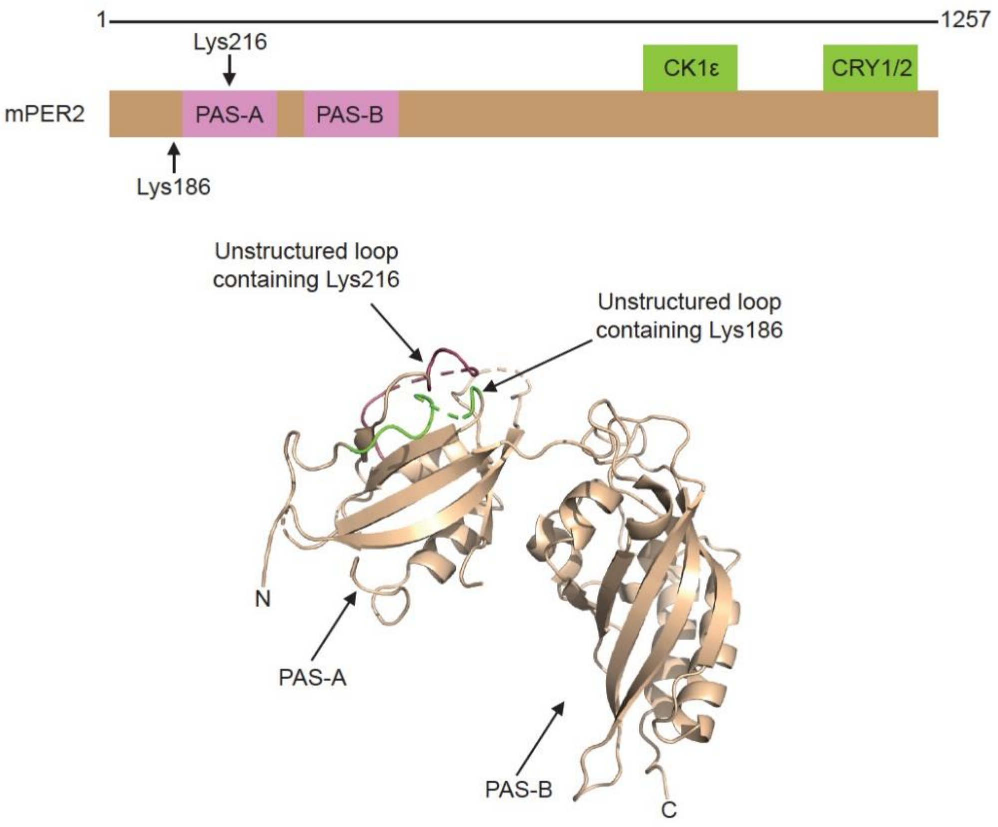

Figure S1. Potential sites for incorporation of amber stop codon in place of lysine in N terminal domain of PERIOD2.

(Upper) Linear cartoon of mouse PER2 illustrating the PAS-A and PAS-B domains and lysine residues along with CK1ε and CRY1/2 binding regions. (Lower) Three-dimensional image of PAS-A and PAS-B domains of PER2 with position of lysine residues in unstructured loops. After (Hennig et al., 2009).

Supplementary Figure 2

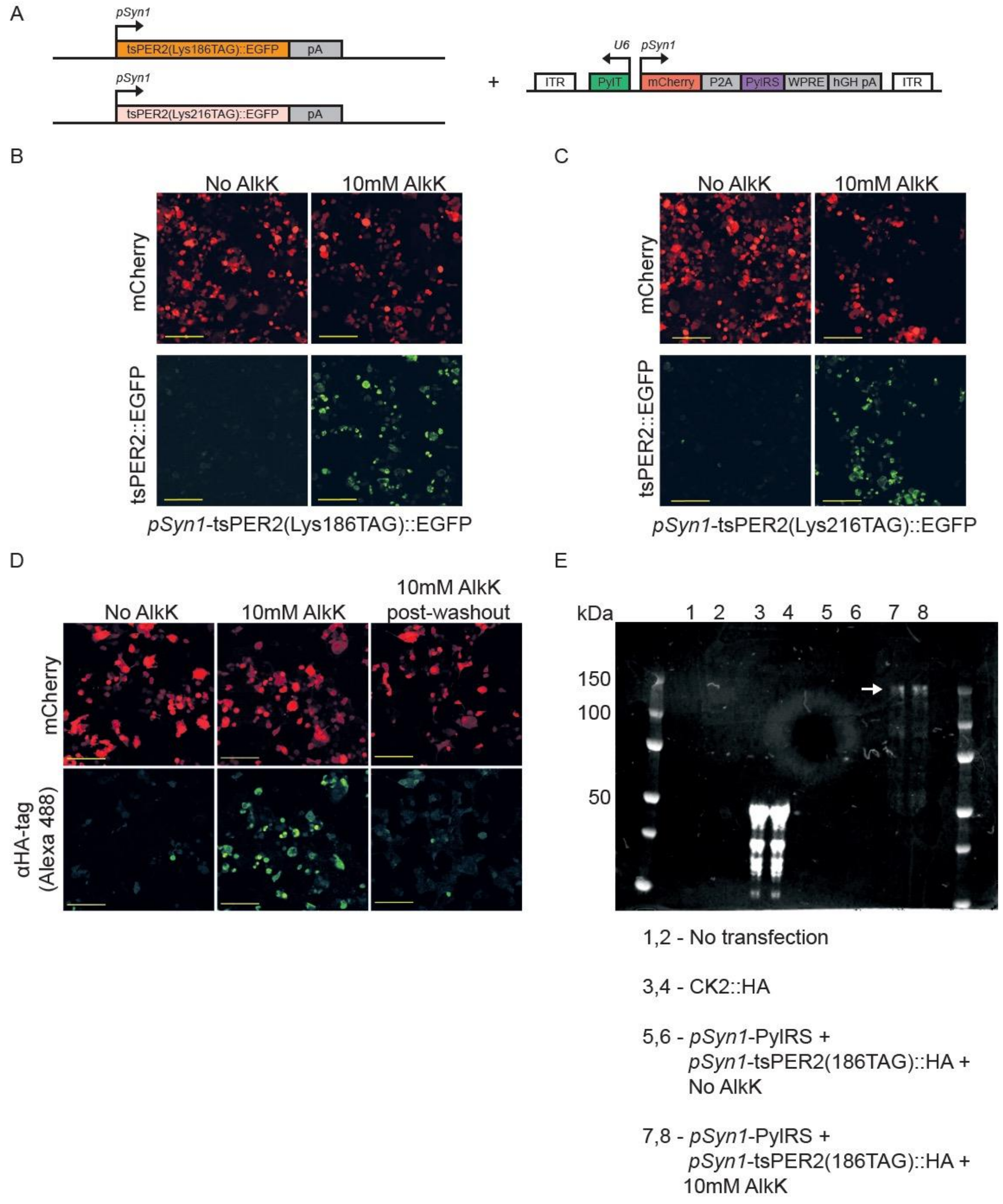

Figure S2. Validation of translational switching to control expression of PER2 in cell culture.

(A) (Left) Schematic of two AAV constructs carrying cDNA encoding PER2 with an amber codon in place of a lysine codon either at position 186 (upper, orange) or position 216 (lower, pink) and a C-terminal EGFP tag, driven by human *Syn1* promoter (*pSyn1*). (Right) Schematic AAV for expression of orthogonal tRNA and complementary tRNA synthetase with

mCherry reporter. PylRS: Pyrrolysyl tRNA synthetase, PylT: tRNAPylCUA: P2A: 2A cleavable peptide: WPRE: Woodchuck Hepatitis Virus (WHV) Posttranscriptional Regulatory Element: hGHpA: growth hormone poly-adenylation site: ITR: viral inverted terminal repeat. (B) Fluorescent images of HEK293T cells co-transfected with the orthogonal tRNA/ tRNA synthetase pair along with *pSyn1*-tsPER2(Lys186TAG)::EGFP. (Upper) mCherry signal from tRNA synthetase plasmid. (Lower) EGFP C-terminal signal from full-length tsPER2. Cells were treated with vehicle (left) or 10 mM AlkK (right). Scale bar is 100 μm. (C) Same as in (B) but for *pSyn1*-tsPER2(Lys216TAG)::EGFP. (D) Fluorescent images of HEK293T cells co-transfected with the orthogonal tRNA/ tRNA synthetase pair along with *pSyn1*-tsPER2(Lys186TAG)::HA. Cells were untreated and imaged (left), treated with 10 mM AlkK and imaged (centre) or treated with AlkK and imaged after wash out (right). (Upper) mCherry signal from tRNA synthetase plasmid. (Lower) Immunostaining for HA tag on tsPER2. Scale bar is 100 μm. (E) Western blot showing expression of tsPER2(Lys186TAG)::HA in HEK293T cells, transfected as in C, only after provision of AlkK. Lanes 1, 2 no transfection; 3, 4 positive control for HA-tag immunostaining (CK2α::HA at 44 kDa); 5, 6 no AlkK, 7, 8 10 mM AlkK, note band a ~130kDa (arrowed) corresponding to tsPER2(Lys186TAG)::HA. Lane 4 positive control shows strong expression of HA-tagged casein kinase 2 in HEK293T under the *CMV* promoter. The expression of tsPER2(186TAG)::HA is lower as it was driven by the Syn1 promoter.

Supplementary Figure 3

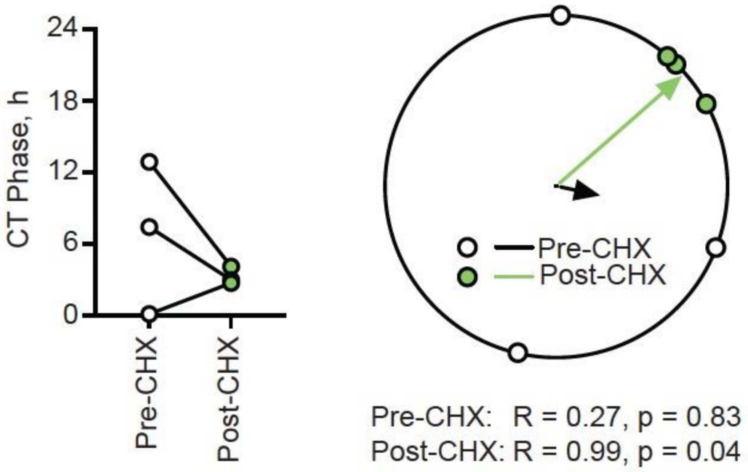

Figure S3. Washout of cycloheximide synchronises PER2::Luciferase SCN organotypic slices to a common phase of oscillation.

(Left) Paired plot of SCN phase before (left, black) and after (right, light green) wash-out of cycloheximide (CHX) as a positive control for SCN phase-resetting. (Right) Circular plot showing that post-CHX washout, slices restart from a common phase as indicated by the vector length of the arrow which represents the Rayleigh statistic (R). R values and their associated p-values are reproduced below the plot.

Supplementary references

Bae, K., Jin, X., Maywood, E. S., Hastings, M. H., Reppert, S. M., & Weaver, D. R. (2001). Differential functions of mPer1, mPer2, and mPer3 in the SCN circadian clock. *Neuron, 30*(2), 525-536. doi:10.1016/s0896-6273(01)00302-6

Chen, T. W., Wardill, T. J., Sun, Y., Pulver, S. R., Renninger, S. L., Baohan, A., . . . Kim, D. S. (2013). Ultrasensitive fluorescent proteins for imaging neuronal activity. *Nature, 499*(7458), 295-300. doi:10.1038/nature12354

Dana, H., Mohar, B., Sun, Y., Narayan, S., Gordus, A., Hasseman, J. P., . . . Kim, D. S. (2016). Sensitive red protein calcium indicators for imaging neural activity. *Elife, 5*. doi:10.7554/eLife.12727

Ernst, R. J., Krogager, T. P., Maywood, E. S., Zanchi, R., Beranek, V., Elliott, T. S., . . . Chin, J. W. (2016). Genetic code expansion in the mouse brain. *Nat Chem Biol, 12*(10), 776-778. doi:10.1038/nchembio.2160

Hennig, S., Strauss, H. M., Vanselow, K., Yildiz, O., Schulze, S., Arens, J., . . . Wolf, E. (2009). Structural and functional analyses of PAS domain interactions of the clock proteins Drosophila PERIOD and mouse PERIOD2. *PLoS Biol, 7*(4), e94. doi:10.1371/journal.pbio.1000094

Maywood, E. S., Drynan, L., Chesham, J. E., Edwards, M. D., Dardente, H., Fustin, J. M., . . . Hastings, M. H. (2013). Analysis of core circadian feedback loop in suprachiasmatic nucleus of mCry1-luc transgenic reporter mouse. *Proc Natl Acad Sci U S A, 110*(23), 9547-9552. doi:10.1073/pnas.1220894110

Yamaguchi, S., Isejima, H., Matsuo, T., Okura, R., Yagita, K., Kobayashi, M., & Okamura, H. (2003). Synchronization of cellular clocks in the suprachiasmatic nucleus. *Science, 302*(5649), 1408-1412. doi:10.1126/science.1089287

Yoo, S. H., Yamazaki, S., Lowrey, P. L., Shimomura, K., Ko, C. H., Buhr, E. D., . . . Takahashi, J. S. (2004). PERIOD2::LUCIFERASE real-time reporting of circadian dynamics reveals persistent circadian oscillations in mouse peripheral tissues. *Proc Natl Acad Sci U S A, 101*(15), 5339-5346. doi:10.1073/pnas.0308709101
